# The impact of COVID-19 on home, social, and productivity integration of people with chronic traumatic brain injury or stroke living in the community

**DOI:** 10.1097/MD.0000000000028695

**Published:** 2022-02-25

**Authors:** Alejandro García-Rudolph, Joan Saurí, Blanca Cegarra, Eloy Opisso, Josep María Tormos, Dietmar Frey, Vince Istvan Madai, Montserrat Bernabeu

**Affiliations:** a Department of Research and Innovation, Institut Guttmann, Institut Universitari de Neurorehabilitació adscrit a la UAB, Badalona, Barcelona, Spain; b Universitat Autònoma de Barcelona, Bellaterra (Cerdanyola del Vallès), Spain; c Fundació Institut d’Investigació en Ciències de la Salut Germans Trias i Pujol, Badalona, Barcelona, Spain; d Charité Lab for Artificial Intelligence in Medicine – CLAIM, Charité - Universitätsmedizin Berlin, Berlin, Germany; e QUEST Center for Transforming Biomedical Research, Berlin Institute of Health (BIH), Charité - Universitätsmedizin Berlin, Berlin, Germany; f School of Computing and Digital Technology, Faculty of Computing, Engineering and the Built Environment, Birmingham City University, Birmingham, United Kingdom.

**Keywords:** community integration, coronavirus disease 2019, stroke, traumatic brain injury

## Abstract

Compare community integration of people with stroke or traumatic brain injury (TBI) living in the community before and during the coronavirus severe acute respiratory syndrome coronavirus 2 disease (COVID-19) when stratifying by injury: participants with stroke (G1) and with TBI (G2); by functional independence in activities of daily living: independent (G3) and dependent (G4); by age: participants younger than 54 (G5) and older than 54 (G6); and by gender: female (G7) and male (G8) participants.

Prospective observational cohort study

In-person follow-up visits (before COVID-19 outbreak) to a rehabilitation hospital in Spain and on-line during COVID-19.

Community dwelling adults (≥18 years) with chronic stroke or TBI.

Community integration questionnaire (CIQ) the total-CIQ as well as the subscale domains (ie, home-CIQ, social-CIQ, productivity CIQ) were compared before and during COVID-19 using the Wilcoxon ranked test or paired *t* test when appropriate reporting Cohen effect sizes (d). The functional independence measure was used to assess functional independence in activities of daily living.

Two hundred four participants, 51.4% with stroke and 48.6% with TBI assessed on-line between June 2020 and April 2021 were compared to their own in-person assessments performed before COVID-19.

When analyzing total-CIQ, G1 (d = −0.231), G2 (d = −0.240), G3 (d = −0.285), G5 (d = −0.276), G6 (d = −0.199), G7 (d = −0.245), and G8 (d = −0.210) significantly decreased their scores during COVID-19, meanwhile G4 was the only group with no significant differences before and during COVID-19.

In productivity-CIQ, G1 (d = −0.197), G4 (d = −0.215), G6 (d = −0.300), and G8 (d = −0.210) significantly increased their scores, meanwhile no significant differences were observed in G2, G3, G5, and G7.

In social-CIQ, all groups significantly decreased their scores: G1 (d = −0.348), G2 (d = −0.372), G3 (d = −0.437), G4 (d = −0.253), G5 (d = −0.394), G6 (d = −0.319), G7 (d = −0.355), and G8 (d = −0.365).

In home-CIQ only G6 (d = −0.229) significantly decreased, no significant differences were observed in any of the other groups.

The largest effect sizes were observed in total-CIQ for G3, in productivity-CIQ for G6, in social-CIQ for G3 and in home-CIQ for G6 (medium effect sizes).

Stratifying participants by injury, functionality, age or gender allowed identifying specific CIQ subtotals where remote support may be provided addressing them.

## 1 Introduction

Stroke is a leading cause of disability worldwide, affecting an increasing number of people at working-age^[1]^ it places special demands on rehabilitation services and re-integration into society.^[2]^ Community re-integration and participation are goals in many policy documents, but also complex processes that have been previously reported to require healthcare professionals’ support.^[3]^

Similarly, traumatic brain injury (TBI) represents the greatest contributor to disability among all trauma-related injuries.^[4]^ Despite the fact that community integration (CI) is a hallmark goal of rehabilitation, there is a notable gap in our understanding of factors that contribute to diminished CI within TBI populations.^[5]^

CI is defined as one's active participation into 3 major areas: home integration as an active participation of the individual in the operations of the home; social integration as participation in a variety of activities outside the home, for example, social events; and productive activities such as employment, and educational and/or volunteer activities.^[6]^

Previous longitudinal studies are consistent in concluding that older persons with TBI had lower CI levels as compared to younger ones. Consistent results were reported at 1 year follow-up^[7–9]^ at 3 years,^[10]^ between 2 and 5 years^[11]^ or between 3 to 15 years.^[12]^ Functional independence was also previously reported as significant predictor of CI at 3 years postinjury^[10]^ or at 1 year.^[13]^ Women appeared to outperform men in overall CI in previous research,^[14]^ for example, in home activities^[12]^ but research addressing CI from a sex perspective is scarce and required.^[15]^

Similarly, in stroke survivors, older individuals have been extensively reported to experience lower levels of integration into the community compared to younger ones in several longitudinal studies.^[16–19]^ Independence in activities of daily living (ADLs) was also found as significant predictor of long-term CI (eg,^[16,20]^). In relation to sex differences, women appear to experience worse functional outcomes^[21]^ and greater participation restrictions^[22]^ with higher prevalence of depression.^[23]^ Ayis et al^[24]^ recently reported that severe depression symptoms in women (up to 5 years poststroke) were double than in men.

The coronavirus disease 2019 (COVID-19)^[25]^ represents nowadays (May 2021) an international health emergency without precedents in terms of its health, economic, and organizational effects on people's lives.^[26]^ Spain has been one of the most affected countries in the world in terms of absolute number of diagnosed cases.^[27]^ On March 13, 2020, the Government declared a national state of alarm, including measures of national lockdown, confinement of the population, and restricted mobility^[28]^ (legally effective on March 15).

Recent research involving general population during COVID-19 pointed out that older people may experience more stress and fear, and that forced isolation may have a severe impact on their psychological well-being.^[29,30]^ Meanwhile other findings suggested that older adults may be able to cope well with the emergency.^[31]^ These conflicting results indicate the need of further research analyzing differences by age. In relation to sex differences, a recent study conducted in Spain concluded that the social impact of COVID-19 on general population was worse in women (33% of women and 17% of men experiencing anxiety and depression)^[32]^ but little is known about sex differences in people with disabilities.

We hypothesized that the identification of specific groups (eg, older participants, individuals with lower level of independence in ADLs) in any of the 3 specific major areas (home integration; social integration; or productive activities) would be useful for providing specialized support interventions remotely conducted by rehabilitation professionals.

Therefore, this study aims at objectively compare CI, considering its 3 major areas (home, social, and productive activities) of people with chronic stroke or TBI living in the community before and during the outbreak of COVID-19. When stratifying by injury: participants with stroke (G1) and with TBI (G2); by functional independence in ADLs: independent (G3) and dependent (G4); by age: participants younger than 54 (G5) and older than 54 (G6); and by gender: female (G7) and male (G8) participants.

## 2 Methods

### 2.1 Study design

We conducted a retrospective observational study enrolling people with stroke or TBI who were living in the community and responded an online assessment. The individuals with stroke or TBI were selected from the electronical health records of a rehabilitation hospital from a Mediterranean setting in Spain (Institut Guttmann - Hospital de Neurorehabilitació - Barcelona). Only participants registered in the hospital's electronical health records with the CIQ (CI questionnaire)^[33]^ assessment previously performed in-person (during a follow-up visit before COVID-19 outbreak) to the hospital's Psychosocial Unit were included in the study. Recruitment period for the online questionnaire was from June 2020 to April 2021.

This study conforms to the Strengthening the Reporting of Observational Studies in Epidemiology Guidelines.^[34]^

The data that support the findings of this study are available from the corresponding author upon reasonable request.

### 2.2 Participants

Eligible participants were living in the community with the diagnosis of stroke or TBI (at the moment of online assessment were aged ≥18), with electronical health records including complete data.

Participants were excluded for the following reasons: diagnosis of concomitant comorbidity (eg, brain tumors), more than 3 years since in-person assessment to COVID-19 lockdown date (March 15, 2020), more than 3 years since in-person functional independence assessment to online CIQ assessment, not fluent in Spanish or other communication issues.

The Psychosocial Unit performs follow-up on CIQ every 3 years. Therefore, every eligible participant was contacted as part of the routine clinical follow-up.

Participants answered their follow-up online assessments within at most 10 days since contacted, therefore all were completed between June 2020 and April 2021. Consequently, participants completed the online measures analyzed in this study as part of a virtual visit involving other assessments (eg, the Craig Handicap Assessment and Reporting Technique.^[35]^

### 2.3 Online assessment

The online assessment was implemented during COVID-19 lockdown in order to provide a remote follow-up service. The online assessment includes the same questionnaires as when participants were assessed in-person (before COVID-19 outbreak) as follow-up visits. Each participant received the online assessment by means of a short message service (SMS) sent to the participant's mobile phone. This SMS is sent by the professional from the psychosocial unit in charge of the participants’ online follow-up. In this work we applied the Spanish validated version of the CIQ.^[36]^ It yields 3 domains which examine home integration (denoted in this work as home-CIQ), social integration (denoted in this work as social-CIQ) and productive activities (denoted in this work as productivity CIQ), as detailed in Table S1, Supplemental Digital Content. This version has been used in previous brain injury research.^[37]^

The CIQ is a recognized measure for examining CI following brain injury and is the most widely used CI measurement tool used in research for people with TBI^[38]^ also extensively used to assess CI in individuals with stroke.^[16]^ Psychometric properties were evaluated for the Spanish-language version of the CIQ. Internal consistency was considered adequate and similar to that found by the authors of the original English version (.70 vs .76).^[33]^ The social integration and productive activity subscales were positively related to similar constructs in the Craig Handicap Assessment and Reporting Technique.^[36]^

### 2.4 Clinical and demographic variables

Demographics (age, sex, years of education, marital status), severity of the injury by means of the National Institutes of Health Stroke Scale^[39]^ and the Glasgow Coma Scale,^[40]^ as well as time since onset of the injury, were collected from the hospital's electronical health records. The functional independence measure (FIM)^[41]^ was used to assess independence in ADLs.

The hospital's interdisciplinary stroke and TBI rehabilitation teams comprise the following professionals with expertise in rehabilitation: neurologist, physiatrist, physiotherapist, occupational therapist, speech and language therapist, neuropsychologist, and nurse. Trained therapists recorded admission and discharge FIM scores, and are systematically reviewed as part of our formal rehabilitation program.

A cutoff value of motor FIM <61 was used to stratify dependent and independent performance in ADLs as in previous research.^[42]^

### 2.5 Statistical analyses

All statistical analyses were performed in R-v4.05 (64 bits) (Vienna, Austria),^[43]^ level of significance was set at *P* = .05. Descriptive statistics were used for demographic and clinical characteristics of participants. Responses to CIQ were compared before COVID-19 outbreak and during it using the Wilcoxon ranked test or paired *t* test when appropriate. The Shapiro–Wilk test was used to assess normality, Levene test for homogeneity of variances and Cohen d to assess effects sizes (small effect size [d = .1], medium [d = .3], and large effect size [d = .5]).

### 2.6 Ethical considerations

A specific written informed consent was not required for participants to be included in this study, in accordance with the local legislation and institutional requirements. Nevertheless at admission participants provide written informed consent to be included in research studies addressed by the hospital. The authors confirm that this study is compliant with the Helsinki Declaration of 1975, as revised in 2008 and it was approved by the Ethics Committee of Clinical Research of the hospital.

## 3 Results

The initial number of eligible participants, considering the procedure described in Section 2.2. was n = 237. Three participants presented another disabling condition (anoxia and brain tumors) and 2 of them were not fluent in Spanish. Two hundred thirty-two received the SMS in their mobile phones, 6 (2.6%) did not complete the CIQ assessment, 18 (7.7%) were assessed in-person more than 3 years before the COVID-19 lockdown date and 4 (1.7%) had more than 3 years since in-person FIM assessment to online CIQ assessment. A detailed flowchart is presented in Figure S1, Supplemental Digital Content.

Consequently, a total of 204 individuals were included in the study, 105 individuals with stroke (51.4%) and 99 with TBI (48.6%).

Table 1 presents their demographics and clinical characteristics. The mean time since injury to online assessment was 9.88 ± 7.02 years for participants with stroke and 13.64 ± 7.04 years for participants with TBI. The mean time since CIQ in-person assessment to CIQ online assessment was 2.00 ± 0.49 and 2.19 ± 0.76 years, respectively. The mean time since lockdown (March 15) to online assessment was 0.67 ± 0.28 years and 0.65 ± 0.29 years, respectively. Participants with stroke were older: 57.46 ± 10.21 and 47.33 ± 11.87, respectively at the moment of online assessment.

**Table 1:** Comparison of demographics and clinical characteristics of individuals with stroke (n = 105) and traumatic brain injury (n = 99).

Variables	Stroke (N = 105)	TBI (N = 99)	*P*
Sex (%)			**.035**
Male	61.0	74.7	
Female	39.0	25.3	
Age at the moment of injury in years, mean (SD)	47.58 (10.19)	33.68 (14.83)	**<.001**
Age at the moment of online assessment, mean (SD)	57.46 (10.21)	47.33 (11.87)	**<.001**
Age <65 at the moment of online assessment (%)	77.1	89.9	**.015**
Age ranges at the moment of online assessment (%)			**<.001**
18–30	1.9	4.0	
31–45	9.5	53.5	
46–60	49.5	25.3	
61–75	37.1	16.2	
76+	1.9	1.0	
Time (in yr) since lockdown (March 14) to CIQ online assessment, mean (SD)	0.67 (0.28)	0.65 (0.29)	.556
Time (in yr) since CIQ in-person assessment to CIQ online assessment, mean (SD)	2.00 (0.49)	2.19 (0.76)	.232
Time (in yr) since in-person CIQ assessment to lockdown (March 14), mean (SD)	1.32 (0.58)	1.54 (0.79)	.076
Time (in yr) since injury to online assessment, mean (SD)	9.88 (7.02)	13.64 (7.04)	**<.001**
Time (range in yr) since injury to online assessment (%)			**<.001**
3–6	42.9	23.2	
7–12	33.3	29.3	
13–18	20.0	22.2	
19+	3.8	25.3	
Time (in yr) since injury to lockdown assessment, mean (SD)	9.20 (7.04)	12.99 (7.06)	**<.001**
Severity at the moment of injury (%)			.642
Mild	12.8	14.6	
Moderately severe and severe	87.2	85.4	
FIM in-person assessment, mean (SD)			
Cognitive FIM	28.61 (7.71)	27.01 (8.65)	.201
Motor FIM	65.81 (22.30)	69.34 (28.41)	**.004**
Total FIM	94.42 (27.94)	96.35 (36.22)	**.032**
Time (in yr) since FIM in-person assessment to the CIQ online assessment, mean (SD)	2.13 (0.70)	2.29 (0.96)	.278
Years of education at the moment of online assessment (%)			.011
Read and write (<2 yr)	9.5	3.0	
Primary (2–5 yr)	27.6	39.4	
Secondary (6–12 yr)	42.9	27.3	
Higher (>13 yr)	20.0	30.3	
Marital status. Married (%)	80.0	34.3	<.001
Location where participants were living at the moment of answering the online assessment (%)			.223
Barcelona	78.1	73.7	
Girona	11.4	9.1	
Tarragona	8.6	9.1	
Lerida	1.9	8.1	
CIQ assessment (in person)			
Home-CIQ	4.06 (3.07)	5.20 (3.64)	.032
Social-CIQ	6.73 (2.16)	6.95 (2.46)	.288
Productivity-CIQ	0.47 (1.11)	1.29 (1.66)	<.001
Total-CIQ	11.26 (4.52)	13.44 (6.67)	.009
CIQ assessment (online)			
Home-CIQ	3.91 (3.17)	4.87 (3.66)	.075
Social-CIQ	5.81 (2.33)	6.11 (2.56)	.274
Productivity-CIQ	0.77 (1.50)	1.63 (1.90)	<.001
Total-CIQ	10.50 (5.12)	12.60 (6.71)	.020

All characteristics are presented as percentages (%), unless otherwise indicated. CIQ = community integration questionnaire, FIM = functional independence measure, SD = standard deviation, TBI = traumatic brain injury. Bold value indicates level of significance was set at *P* = .05

### 3.1 Stratification by injury: participants with stroke

Table 2 presents comparisons before and during COVID-19 for individuals with stroke (G1), (n = 105, 51.4%)

**Table 2:** Paired comparisons for pre-COVID assessments and during COVID for participants with stroke (n = 105) (G1).

	COVID	Median	Mean (SD)	S–W test (*P*)	Wilcoxon test/*t* test (*P*)	Effects sizes (d)
Groceries	Before	1.00	1.30 (0.71)	<.001	.199	−0.125
	During	1.00	1.23 (0.76)	<.001		
Prepare meals	Before	0.00	0.66 (0.88)	<.001	.636	−0.046
	During	0.00	0.63 (0.84)	<.001		
Housework	Before	0.00	0.66 (0.81)	<.001	**.0356**	−**0.205**
	During	0.00	0.55 (0.71)	<.001		
Plans social	Before	1.00	0.80 (0.75)	<.001	.324	−0.092
	During	1.00	0.86 (0.80)	<.001		
Personal finances	Before	0.00	0.64 (0.81)	<.001	.832	−0.0206
	During	0.00	0.65 (0.82)	<.001		
Home-CIQ	Before	4.00	4.06 (3.07)	<.001	.197	−0.125
	During	3.00	3.91 (3.17)	<.001		
Leisure activities	Before	1.00	1.10 (0.63)	<.001	**<.001**	−**0.358**
	During	1.00	0.84 (0.59)	<.001		
Visit friends	Before	1.00	1.12 (0.63)	<.001	**.004**	−**0.275**
	During	1.00	0.93 (0.64)	<.001		
Leisure with others	Before	2.00	1.48 (0.65)	<.001	.368	−0.087
	During	2.00	1.41 (0.68)	<.001		
Best friend	Before	2.00	1.24 (0.98)	<.001	.245	−0.113
	During	2.00	1.12 (1.00)	<.001		
Travel out home	Before	2.00	1.79 (0.47)	<.001	**<.001**	−**0.388**
	During	2.00	1.50 (0.72)	<.001		
Social-CIQ	Before	7.00	6.73 (2.16)	<.001	**<.001**	−**0.348**
	During	6.00	5.81 (2.33)	<.001		
Work situation	Before	0.00	0.26 (0.52)	<.001	.344	−0.092
	During	0.00	0.21 (0.43)	<.001		
Training situation	Before	0.00	0.09 (0.28)	<.001	**.012**	−**0.243**
	During	0.00	0.19 (0.42)	<.001		
Volunteer situation	Before	0.00	0.09 (0.37)	<.001	.849	−0.018
	During	0.00	0.10 (0.35)	<.001		
Productivity-CIQ	Before	0.00	0.47 (1.11)	<.001	**.043**	−**0.197**
	During	0.00	0.77 (1.50)	<.001		
Total-CIQ^∗^	Before	12.00	11.26 (4.52)	.190	**.017** ^∗∗^	−**0.231**
	During	10.00	10.50 (5.12)	.230		

CIQ = community integration questionnaire, COVID = coronavirus disease, SD = standard deviation, S–W test = Shapiro–Wilk test.

∗ Levene test *P* = .622 the variances are not significantly different, indicating that the assumption of homogeneity has been met.

∗∗ *t* test: *t* = 2.406, df = 104, *P*-value = .01789. Bold value indicates level of significance was set at *P* = .05

For G1 in this section and for G2 in the next section we compared home-CIQ, social-CIQ, productivity-CIQ, and total-CIQ but we also compared all CIQ individual items comprising each domain. Nevertheless, individual items were not reported to psychometrically measure any valid or reliable construct in the original English version^[33]^ neither in the Spanish validated version.^[36]^ Therefore the specific items comparisons are included in Tables 2 and 3 only as additional information.

**Table 3:** Paired comparisons for pre-COVID assessments and during COVID for participants with traumatic brain injury (n = 99) (G2).

	COVID	Median	Mean (SD)	S–W test (*P*)	Wilcoxon test/*t* test (*P*)	Effects sizes (d)
Groceries	Before	2.00	1.47 (0.79)	<.001	**.012**	**−0.243**
	During	2.00	1.36 (0.81)	<.001		
Prepare meals	Before	1.00	1.05 (0.93)	<.001	.437	**−**0.075
	During	1.00	1.00 (0.93)	<.001		
Housework	Before	1.00	1.03 (0.86)	<.001	**.001**	**−0.311**
	During	1.00	0.82 (0.85)	<.001		
Plans social	Before	1.00	0.94 (0.83)	<.001	.759	**−**0.029
	During	1.00	0.96 (0.84)	<.001		
Personal finances	Before	0.00	0.71 (0.85)	<.001	.718	**−**0.035
	During	0.00	0.73 (0.85)	<.001		
Home-CIQ	Before	6.00	5.20 (3.64)	<.001	.112	**−**0.154
	During	5.00	4.87 (3.66)	<.001		
Leisure activities	Before	1.00	1.13 (0.65)	<.001	.057	**−**0.185
	During	1.00	0.99 (0.63)	<.001		
Visit friends	Before	1.00	1.26 (0.68)	<.001	**.002**	**−0.296**
	During	1.00	1.02 (0.64)	<.001		
Leisure with others	Before	2.00	1.60 (0.57)	<.001	.057	**−**0.185
	During	2.00	1.47 (0.63)	<.001		
Best friend	Before	2.00	1.21 (0.98)	<.001	.324	**−**0.096
	During	2.00	1.11 (1.00)	<.001		
Travel out home	Before	2.00	1.75 (0.59)	<.001	**.003**	**−0.283**
	During	2.00	1.52 (0.80)	<.001		
Social-CIQ	Before	7.00	6.95 (2.46)	<.001	**<.001**	**−0.372**
	During	7.00	6.11 (2.56)	<.001		
Work situation	Before	0.00	0.63 (0.82)	<.001	.946	**−**0.001
	During	0.00	0.62 (0.83)	<.001		
Training situation	Before	0.00	0.28 (0.50)	<.001	1.00	**−**
	During	0.00	0.28 (0.50)	<.001		
Volunteer situation	Before	0.00	0.16 (0.49)	<.001	.490	**−**0.060
	During	0.00	0.20 (0.51)	<.001		
Productivity-CIQ	Before	0.00	1.29 (1.66)	<.001	.077	**−**0.172
	During	0.00	1.63 (1.90)	<.001		
Total CIQ	Before	15.00	13.44 (6.68)	<.001	**.013**	**−0.240**
	During	14.00	12.61 (6.71)	<.001		

CIQ = community integration questionnaire, COVID = coronavirus disease, SD = standard deviation, S–W test = Shapiro–Wilk test. Bold value indicates level of significance was set at *P* = .05

No significant differences were observed in home-CIQ, meanwhile social-CIQ significantly decreased (d = −0.348), productivity-CIQ significantly increased (d = −0.197), and total-CIQ significantly decreased (d = −0.231).

The specific item with the highest effect size was travel out home (d = −0.388).

### 3.2 Stratification by injury: participants with TBI

Table 3 presents comparisons before and during COVID-19 for individuals with TBI (G2), (n = 99, 48.6%).

No significant differences were observed in home-CIQ neither in productivity-CIQ, meanwhile social-CIQ significantly decreased (d = −0.372) as well as total-CIQ (d = −0.240).

The specific item with the highest effect size was housework (d = −0.311).

### 3.3 Stratification by functional independence, age, and sex

Table 4 presents comparisons before and during COVID-19 for independent participants (G3), (n = 144, 70.6%), dependent participants (G4) (n = 60, 29.4%), participants younger than 54 (G5) (n = 111, 54.4%), older than 55 (G6) (n = 93, 45.6%), women (G7) (n = 66, 32.4%), and men (G8) (n = 138, 67.6%).

**Table 4:** Paired comparisons for pre-COVID and during COVID assessments.

Group	Measures	COVID	Median	Mean (SD)	S–W test (*P*)	Wilcoxon test/*t* test (*P*)	Effects sizes (d)
Motor FIM > 61 (n = 144) (G3)	Home-CIQ	Before	6.00	6.08 (2.84)	<.001	.066	−0.179
		During	6.00	5.82 (2.99)	<.001		
	Social-CIQ	Before	8.00	7.29 (2.07)	<.001	**<.001**	−**0.437**
		During	7.00	6.42 (2.33)	<.001		
	Productivity-CIQ	Before	0.00	1.11 (1.57)	<.001	.055	−0.187
		During	0.00	1.39 (1.81)	<.001		
	Total-CIQ	Before	14.00	14.47 (5.01)	.024	**.003**	−**0.285**
		During	14.00	13.64 (5.42)	.040		
Motor FIM ≤ 61 (n = 60) (G4)	Home-CIQ	Before	1.00	1.10 (1.56)	<.001	.477	−0.069
		During	0.00	0.90 (1.28)	<.001		
	Social-CIQ	Before	6.00	5.75 (2.49)	<.001	**.009**	−**0.253**
		During	5.00	4.83 (2.37)	<.001		
	Productivity-CIQ	Before	0.00	0.28 (0.94)	<.001	**.0273**	−**0.215**
		During	0.00	0.70 (1.53)	<.001		
	Total-CIQ	Before	7.00	7.13 (3.85)	.060	.155	−0.189
		During	6.00	6.43 (4.05)	.003		
Age ≤ 54 (n = 111) (G5)	Home-CIQ	Before	6.00	5.21 (3.58)	<.001	.524	−0.064
		During	5.00	5.07 (3.61)	<.001		
	Social-CIQ	Before	8.00	7.17 (2.37)	<.001	**<.001**	−**0.394**
		During	7.00	6.29 (2.63)	<.001		
	Productivity-CIQ	Before	0.00	1.21 (1.64)	<.001	.328	−0.095
		During	0.00	1.37 (1.81)	<.001		
	Total-CIQ	Before	14.00	13.59 (6.20)	.003	**.004**	−**0.276**
		During	14.00	12.73 (6.48)	<.001		
Age ≥55 (n = 93) (G6)	Home-CIQ	Before	4.00	3.90 (3.04)	<.001	**.018**	−**0.229**
		During	3.00	3.55 (3.04)	<.001		
	Social-CIQ	Before	7.00	6.44 (2.16)	<.001	**.002**	−**0.319**
		During	6.00	5.56 (2.15)	<.001		
	Productivity-CIQ	Before	0.00	0.46 (1.09)	<.001	**.002**	−**0.300**
		During	0.00	0.97 (1.67)	<.001		
	Total-CIQ	Before	11.00	10.81 (4.81)	.125	**.041**	−**0.199**
		During	10.00	10.08 (5.10)	.227		
Female (n = 66) (G7)	Home-CIQ	Before	5.50	5.02 (3.52)	<.001	.126	−0.149
		During	5.00	4.70 (3.49)	<.001		
	Social-CIQ	Before	8.00	7.21 (2.14)	<.001	**<.001**	−**0.355**
		During	6.00	6.02 (2.33)	<.001		
	Productivity-CIQ	Before	0.00	0.89 (1.44)	<.001	.098	−0.161
		During	0.00	1.27 (1.76)	<.001		
	Total-CIQ	Before	14.00	13.12 (5.65)	.215	**.0117**	−**0.245**
		During	13.00	11.98 (5.85)	.092		
Male (n = 138)							
(G8)	Home-CIQ	Before	4.00	4.42 (3.33)	<.001	.161	−0.136
		During	4.00	4.22 (3.42)	<.001		
	Social-CIQ	Before	7.00	6.66 (2.36)	<.001	**<.001**	−**0.365**
		During	6.00	5.93 (2.50)	<.001		
	Productivity-CIQ	Before	0.00	0.86 (1.48)	<.001	**.030**	−**0.210**
		During	0.00	1.14 (1.76)	<.001		
	Total-CIQ	Before	12.00	11.93 (5.80)	.009	**.015**	−**0.236**
		During	11.00	11.30 (6.11)	.002		

CIQ = community integration questionnaire, COVID = coronavirus disease, FIM = functional independence measure, SD = standard deviation, S–W test = Shapiro–Wilk test. Bold value indicates level of significance was set at *P* = .05

In G3 no significant differences were observed in home-CIQ neither in productivity-CIQ, meanwhile social-CIQ significantly decreased (d = −0.437) as well as total-CIQ (d = −0.285).

In G4 no significant differences were observed in home-CIQ neither in total-CIQ, meanwhile social-CIQ significantly decreased (d = −0.253) and productivity-CIQ significantly increased (d = −0.215).

In G5 no significant differences were observed in home-CIQ neither in productivity-CIQ, meanwhile social-CIQ significantly decreased (d = −0.394) as well as total-CIQ (d = −0.276).

In G6 home-CIQ significantly decreased (d = −0.229), as well as social-CIQ (d = −0.319) and total-CIQ (d = −0.199), meanwhile productivity-CIQ significantly increased (d = −0.300).

In G7 no significant differences were observed in home-CIQ, neither in productivity-CIQ, meanwhile social-CIQ significantly decreased (d = −0.355) as well as total-CIQ (d = −0.245).

In G8 no significant differences were observed in home-CIQ, meanwhile social-CIQ significantly decreased (d = −0.365), as well as total-CIQ (d = −0.236) and productivity-CIQ significantly increased (d = −0.210).

**Figure 1:**
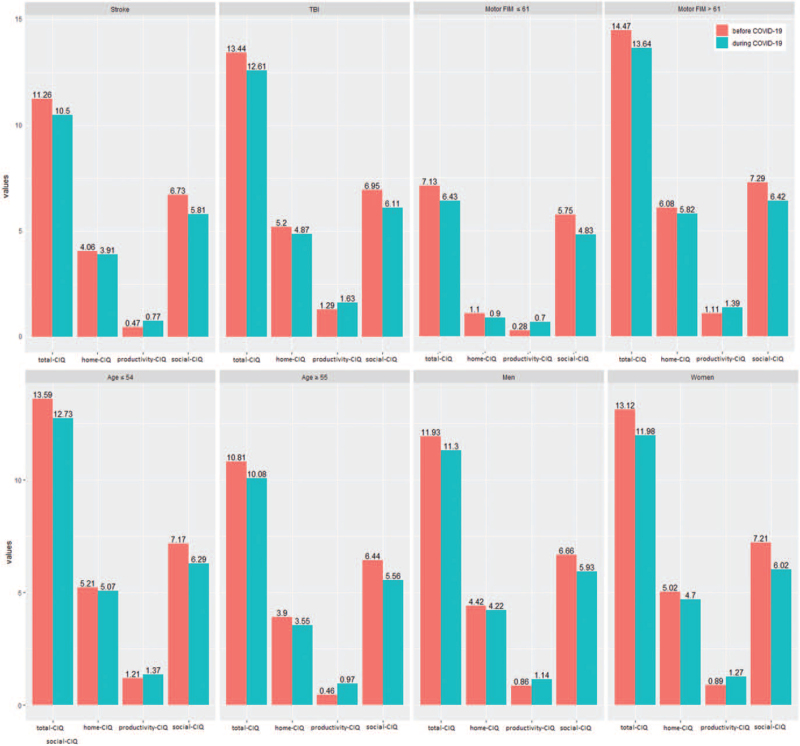
Community integration questionnaire subtotals and total for each stratification (by injury, functional independence, age, and sex) before and during COVID-19. COVID-19 = coronavirus disease 2019.

Table 5 presents the summarization of results considering all stratifications, by injury (G1 and G2), functional independence (G3 and G4), age (G5 and G6), and sex (G7 and G8), also presented as bar graphs in Figure 1.

**Table 5:** Summarization of results for pre-COVID and during COVID assessments.

Stratification	Group	Home-CIQ	Social-CIQ	Productivity-CIQ	Total-CIQ
Injury	Stroke (G1)	=	↓	↑	↓
	TBI (G2)	=	↓	=	↓
Functionality	Motor FIM > 61 points (G3)	=	↓	=	↓
	Motor FIM ≤ 61 points (G4)	=	↓	↑	=
Age	≤ 54 years (G5)	=	↓	=	↓
	≥55 years (G6)	↓	↓	↑	↓
Sex	Female (G7)	=	↓	=	↓
	Male (G8)	=	↓	↑	↓

CIQ = community integration questionnaire, COVID = coronavirus disease, FIM = functional independence measure, TBI = traumatic brain injury. ↓ indicates that the item significantly decreased during COVID. ↑ indicates that the item significantly increased during COVID. = indicates that no significant differences were found when comparing the (sub)total before and during COVID.

## 4 Discussion

Given that the literature concerning COVID-19 is emerging, to our best knowledge no authors have addressed the virus’ impact from a CI perspective addressing home, social and productivity activities on people with chronic stroke or TBI living in the community. We hypothesized that the identification of specific groups (eg, older participants, individuals with lower level of independence in ADLs) in any of the 3 specific major areas (home integration, social integration, or productive activities) would be useful to specialized support interventions remotely conducted by rehabilitation professionals.

We found that the older age group (G6) significantly reduced their participation in home activities, being this the only group who did. We also found that this older group significantly increased their productivity activities, as well as the individuals with lower level of independence (G4), men (G8), and individuals with stroke (G1). Meanwhile, the younger group (G5), individuals with TBI (G2), participants who were independent in ADLs (G3) and women (G7) did not increase their productivity during COVID-19.

Remarkably, the cutoff value of 55 years old is also observed in men's median age, it was 55 (44–64) years, (as presented in Table S2, Supplemental Digital Content) meanwhile women's was 49 (42–57). Median age of participants with motor FIM <61 was also 55 (44–64) meanwhile median age was 52 (43–60) for those with motor FIM >61.

Median age of participants with stroke was 57 (51–64) and for participants with TBI was 44 (39–54).

In relation to young participants, previous studies from nonpandemic times reported that young adults with stroke encounter substantially greater difficulties reintegrating into their social roles compared to older adults.^[44]^ A study including only women aged 18 to 50 years old has also highlighted the young stroke survivors’ unmet needs, including a lack of access to age-appropriate information, inadequate professional support regarding life skills training, limited dialogue with healthcare professionals and underuse of peer learning to support the continual reintegration process.^[45]^ Besides, women's mental health seemed to be more affected when economic conditions worsened during the pandemic. Also, women reported greater concern for their personal finances.^[32]^ Our results, showing a nonsignificant increase in their productivity scores, suggest a remote timely age-appropriate (directly mostly to younger women) provision of educational or training information. In Table S2, Supplemental Digital Content we compared men and women in relation to home-CIQ, social-CIQ, productivity-CIQ, and total-CIQ showing no significant differences between them.

This lack of support seems not to be related to the time since injury, a qualitative study of stroke survivors who were aged 20 to 61 years old and up to 9 years after stroke (in our case it was 9.88 ± 7.02 years) reported their inability to fulfil their role expectations and feelings of isolation or helplessness. Similarly for women with TBI, as reported in previous research, health service providers and policymakers should recognize the long-term health and social needs of women with TBI and address societal factors that result in financial and structural barriers, to ensure access to needed services.^[46]^

A recent survey^[47]^ conducted during the ongoing COVID-19 pandemic on 47 individuals in the chronic phase of moderate–severe TBI, concluded that healthcare providers should look for ways to provide tailored education and reduce social isolation for individuals with disability. They discuss a number of direct suggestions from participant responses.

Pisano et al^[48]^ recently analyzed COVID-19 impact on individuals with aphasia, remarking that global attention is currently focused on clinical populations who, prior to COVID-19 were followed-up in rehabilitation services, but the importance of also considering the individuals with chronic TBI or stroke living in the community needs to be highlighted.

The Spanish Government recently reported the largest study in relation to the impact of COVID-19 on people with disabilities in Spain.^[49]^ The main needs and difficulties experienced by Spanish people with disabilities and their families were reported and concluded that the pandemic had a negative impact in all of the main 5 analyzed areas (employment, education, health, social services, and other basic rights). These results are in accordance with ours. We further analyzed the impact on all CIQ individual items, as well as stratified the participants considering for example their level of functional independence in ADLs.

In our results, G4 was the only group with no significant differences in total-CIQ before and during COVID-19. Their situation means living with several restrictions on participation with or without pandemic situation. In this sense, the figure of the personal assistant can be recommended as a facilitator of empowerment, independent living, and the promotion of community participation.^[50]^

G3 significantly decreased their total-CIQ with the highest effect size, also showing the largest social-CIQ decrease. As mindfulness training has been recently reported to play a key role in alleviating the negative impact related to an individual's boredom proneness, intervention with home-based mindfulness training might be specially beneficial to them.^[51]^

G5 did not significantly increase productivity-CIQ meanwhile G6 not only significantly increased their productivity, they were the group with the highest effect size. Therefore, productivity-related psychological interventions can be recommended to G5. For example, self-regulation, which integrates management of internal (ie, attention, emotion, motivation) and external (ie, environment, support, time) factors. Forms of self-regulation interventions include cognitive-behavior therapy, coherence therapy, and acceptance-based behavior therapy.^[52]^

For G6 self-affirmation theory interventions can be remotely applied in order to promote motivation through maintaining positive perceptions of one's competence and identity.^[53]^

G7 have not significantly increased productivity-CIQ meanwhile G8 did. Recent findings have established an increase in procrastination among students in higher education during COVID-19.^[54]^ Additionally, the nature of online learning from home further encourages procrastination as students not only need to exert higher levels of self-control to overcome isolated learning and the challenges of online learning,^[55]^ they must also resist distractions present at home (eg, television and social media). Furthermore, the increase in procrastination may be attributed to the heightened levels of uncertainty in the pandemic.^[54]^ In these cases, remote Reality Therapy based interventions can be recommended. Reality therapy helps individuals understand and accept that they are responsible for the consequences of their choices.^[56]^ Psycho-educational training sessions that are based upon reality therapy concepts have been proven to be an effective way to reduce academic procrastination behavior^[57]^ shown effective in women with disabilities.^[58]^

### 4.1 Limitations to generalizability and future directions

The data was collected from individuals living in the community but who had been previously undertaken rehabilitation in 1 single tertiary center from a restricted geographical location (Catalonia, with more than 70% from Barcelona). Therefore the generalization of these results should be considered carefully. Nevertheless, assessments by means of standardized tools (CIQ, FIM) allow for similar comparative studies and the restricted physical locations allowed for controlled variability in regional pandemic circumstances.

Given that the pandemic is evolving, there will also be need for ongoing surveillance as to how to support individuals with stroke or TBI at each stage and across additional geographic regions.

Male gender accounts for 61% of participants with stroke and 74% of participants with TBI, suggesting a sex bias, nevertheless, the proportion is similar to recent studies in similar settings.^[42]^

Several cons have been reported^[59]^ regarding online assessments such as: the researcher cannot determine questionnaire filling time and participants may abandon the survey giving partial data; the participant can take his own time to fill form; it may create bias; if the participants have a doubt, researcher cannot clear it immediately. In our case, participants already knew the questionnaires, because they have already answered them during previous in-person follow-up visits.

Another limitation to mention is that in this work we did not use the revised CIQ version which includes an added subscale that assesses use of social media to integrate virtually,^[60]^ which would have been quite useful given the stay at home requirements imposed during COVID-19. Nevertheless this revised version was recently published (2016) and to our best knowledge there is not a validated Spanish version of it.

Future work includes a stratification of stroke participants on those with and without aphasia for comparison with Pisano et al.^[48]^ Further future analysis will address the impact of other social factors (eg, finances, family support, educational level). As shown in Table S2, Supplemental Digital Content 40.9% of women completed high education meanwhile only 17.4% of men did. In spite of women's higher educational level there were no significant differences in productive-CIQ between men and women before COVID-19 neither during it (as shown in Table S2, Supplemental Digital Content), but men significantly increased their productive-CIQ score during COVID-19 meanwhile women didn’t, suggesting future further analysis in relation to such factors.

## 5 Conclusions

We analyzed for the first time the virus’ impact from a CI perspective addressing home, social and productivity activities on people with chronic stroke or TBI living in the community. Stratifying participants by injury, functionality, age or gender allowed identifying specific highly impacted CIQ subtotals and items where remote specialized support can be provided to address them.

## Author contributions

**Conceptualization:** Joan Sauri, Blanca Cegarra, Josep Maria Tormos.

**Data curation:** Alejandro Garcia-Rudolph.

**Formal analysis:** Alejandro Garcia-Rudolph.

**Funding acquisition:** Dietmar Frey, Vince Istvan Madai.

**Investigation:** Alejandro Garcia-Rudolph, Eloy Opisso.

**Methodology:** Alejandro Garcia-Rudolph, Joan Sauri, Blanca Cegarra.

**Project administration:** Eloy Opisso, Josep Maria Tormos, Dietmar Frey, Vince Istvan Madai, Montserrat Bernabeu.

**Resources:** Eloy Opisso.

**Software:** Alejandro Garcia-Rudolph, Eloy Opisso.

**Supervision:** Joan Sauri, Eloy Opisso, Josep Maria Tormos, Vince Istvan Madai, Montserrat Bernabeu.

**Validation:** Blanca Cegarra.

**Visualization:** Blanca Cegarra, Eloy Opisso.

**Writing – original draft:** Alejandro Garcia-Rudolph.

**Writing – review & editing:** Alejandro Garcia-Rudolph, Blanca Cegarra, Eloy Opisso, Josep Maria Tormos, Dietmar Frey, Vince Istvan Madai, Montserrat Bernabeu.

## Supplementary Material

Supplemental Digital Content

## Supplementary Material

Supplemental Digital Content

## Supplementary Material

Supplemental Digital Content
