# Supplementary material for: The impact of COVID-19 on home, social, and productivity integration of people with chronic traumatic brain injury or stroke living in the community
Source: Medicine (Baltimore). 2022 Feb 25;101(8):e28695. doi: 10.1097/MD.0000000000028695 (PMC8878630; doi:10.1097/MD.0000000000028695)
Supplement: Supplemental Digital Content [file medi-101-e28695-s001.docx]

Table SM1. CIQ ítems and domains

| **Id** | **Item** | **Item Spanish** |  |
| --- | --- | --- | --- |
| 1 | Who usually does the shopping for groceries or other necessities in your household? (Groceries) | ¿Quién hace normalmente las compras de alimentos y otras necesidades en su casa? | Home-CIQ |
| 2 | Who usually prepares meals in your household? (Prepares meals) | ¿Quién prepara normalmente los alimentos en su casa? |  |
| 3 | In your home, who usually does normal everyday housework? (Housework) | ¿Quién hace normalmente el trabajo de la casa? |  |
| 4 | Who usually plans social arrangements such as get-togethers with family and  friends? (Plans social) | ¿Quién hace normalmente los planes sociales con amigos y familiares? |  |
| 5 | Who usually looks after your personal finances such as banking or paying bills? (Personal finances) | ¿Quién cuida normalmente sus finanzas personales como el banco o pagar cuenta? |  |
| 6 | Can you tell me approximately how many times a month you now usually participate in the following activities outside your home?  Leisure activities such as movies, sports, restaurants (Leisure activities) | ¿Puede usted decirme aproximadamente, cuantas veces al mes participa en las  siguientes actividades fuera de su casa?  Actividades de tiempo libre como cine, deportes, restaurantes | Social-CIQ |
| 7 | Can you tell me approximately how many times a month you now usually participate in the following activities outside your home?  Visiting friends or relatives (Visit friends) | ¿Puede usted decirme aproximadamente, cuantas veces al mes participa en las siguientes actividades fuera de su casa?  Visitando amigos(as) o familiares |  |
| 8 | When you participate in leisure activities, do you usually do this alone or with others? (Leisure with others) | ¿Cuando participa en actividades de tiempo libre, lo hace solo o acompañado? |  |
| 9 | Do you have a best friend with whom you confide? (Best friend) | ¿Tiene usted un mejor amigo en quien puede confiar? |  |
| 10 | How often do you travel outside the home?  (Travel outside home) | ¿Con que´ frecuencia viaja a fuera de la casa? |  |
| 11 | Current (during the past month) work situation  (Work situation) | Situación laboral (durante el mes pasado) | Productivity-CIQ |
| 12 | Current (during the past month) school or training program situation (Training situation) | Escuela o programa de entrenamiento (durante el mes pasado |  |
| 13 | In the past month, how often did you engage in volunteer activities? (Volunteer activities) | ¿Durante el mes pasado, cuántas veces participó en actividades voluntarias? |  |
