# Supplementary material for: The impact of COVID-19 on home, social, and productivity integration of people with chronic traumatic brain injury or stroke living in the community
Source: Medicine (Baltimore). 2022 Feb 25;101(8):e28695. doi: 10.1097/MD.0000000000028695 (PMC8878630; doi:10.1097/MD.0000000000028695)
Supplement: Supplemental Digital Content [file medi-101-e28695-s002.docx]

Figure SM1. Participant’s selection flowchart

Identification

Eligibility

Included

Screening

Not completed the online CIQ assessment (n= 6)

More than 3 years since in-person FIM assessment to online CIQ assessment (n=4)

Records after initial constraints, who received the SMS in their mobile phones
(n =232)

Another disabling condition (anoxia, brain tumors (n= 3)

Not fluent in Spanish (n=2)

Records identified through database searching with primary diagnosis of stroke or TBI having in-person CIQ assessment

(n =237)

Records after quality constraints applied at admission

(n =222)

Records included for analysis
(n =204)

In-person assessments performed more than 3 years before the COVID-19 lockdown date

(n=18)
