# Supplementary material for: The impact of COVID-19 on home, social, and productivity integration of people with chronic traumatic brain injury or stroke living in the community
Source: Medicine (Baltimore). 2022 Feb 25;101(8):e28695. doi: 10.1097/MD.0000000000028695 (PMC8878630; doi:10.1097/MD.0000000000028695)
Supplement: Supplemental Digital Content [file medi-101-e28695-s003.docx]

Table SM2. Comparison of demographics and clinical characteristics of men (n=138) and women (n=66)

| **Variables** | **Men**  **(N = 138)** | **Women**  **(N=66)** | **p** |
| --- | --- | --- | --- |
| **Injury (%)** |  |  | **0.035** |
| Stroke | 46.4 | 62.1 |  |
| TBI | 53.6 | 37.9 |  |
| **Age at the moment of injury in years,**  **Mean (SD)** | 41.37 (15.09) | 39.72 (12.93) | 0.329 |
| **Age at the moment of online assessment, Mean(SD)** | 53.42 (12.75) | 50.70 (10.60) | 0.092 |
| **Age at the moment of online assessment, Median(Q1-Q3)** | 55 (44- 64) | 49 (42-57) |  |
| **Age < 65 at the moment of online assessment (%)** | 79.7 | 90.9 | **0.045** |
| **Age ranges at the moment of online assessment (%)** |  |  | 0.297 |
| 18-30 | 2.9 | 3.0 |  |
| 31-45 | 30.4 | 31.8 |  |
| 46-60 | 34.1 | 45.5 |  |
| 61-75 | 30.4 | 19.7 |  |
| 76+ | 2.2 | 0.0 |  |
| **Time (in years) since lockdown (March 15^th^) to CIQ online assessment, Mean (SD)** | 0.65 (0.29) | 0.68 (0.27) | 0.452 |
| **Time (in years) since CIQ in-person assessment to CIQ online assessment, Mean (SD)** | 2.10 (0.68) | 2.07 (0.54) | 0.472 |
| **Time (in years) since in-person CIQ assessment to lockdown (March 15^th^), Mean (SD)** | 1.45 (0.74) | 1.38 (0.59) | 0.577 |
| **Time (in years) since injury to online assessment, Mean(SD)** | 12.05 (7.86) | 10.98 (5.80) | 0.557 |
| **Time (range in years) since injury to online assessment (%)** |  |  | 0.160 |
| 3 - 6 | 31.2 | 37.9 |  |
| 7-12 | 34.1 | 25.8 |  |
| 13-18 | 18.1 | 27.3 |  |
| 19+ | 16.7 | 9.1 |  |
| **Time (in years) since injury to lockdown assessment, Mean(SD)** | 11.40 (7.88) | 10.29 (5.84) | 0.515 |
| **Severity at the moment of injury (%)** |  |  | 0.642 |
| Mild | 12.8 | 14.6 |  |
| Moderately Severe and Severe | 87.2 | 85.4 |  |
| **FIM in-person assessment, Mean(SD)** |  |  |  |
| Cognitive FIM | 28.22 (7.41) | 27.03(9.65) | 0.954 |
| Motor FIM | 69.08 (24.30) | 64.25 (27.59) | 0.222 |
| Total FIM | 97.31 (30.24) | 91.28 (35.74) | 0.329 |
| **Time in years since FIM in-person assessment to the CIQ online assessment, Mean(SD)** | 2.15 (0.77) | 2.32 (0.96) | 0.176 |
| **Years of education at the moment of online assessment (%)** |  |  | **<0.001** |
| Read and write (< 2 years) | 8.0 | 3.0 |  |
| Primary (2-5 years) | 49.6 | 18.2 |  |
| Secondary (6-12 years) | 34.1 | 37.9 |  |
| Higher (> 13 years) | 17.4 | 40.9 |  |
| **Marital status. Married (%)** | 58.7 | 56.1 | 0.286 |
| **Location where participants were living at the moment of answering the online assessment(%)** |  |  | 0.865 |
| Barcelona | 75.4 | 77.3 |  |
| Girona | 10.1 | 10.6 |  |
| Tarragona | 8.7 | 9.1 |  |
| Lerida | 5.8 | 3.0 |  |
| **CIQ assessment (in person)** |  |  |  |
| home-CIQ | 4.42 (3.33) | 5.01 (3.51) | 0.324 |
| social-CIQ | 6.65 (2.36) | 7.21 (2.14) | 0.113 |
| productivity-CIQ | 0.85 (1.47) | 0.89 (1.43) | 0.755 |
| total-CIQ | 11.93 (5.79) | 13.12 (5.65) | 0.168 |
| **CIQ assessment (online)** |  |  |  |
| home-CIQ | 4.22 (3.42) | 4.69 (3.48) | 0.390 |
| social-CIQ | 5.92 (2.50) | 6.01 (2.33) | 0.905 |
| productivity-CIQ | 1.14 (1.75) | 1.27 (1.75) | 0.534 |
| total-CIQ | 11.29 (6.11) | 11.98 (5.85) | 0.376 |

All characteristics are presented as percentages (%), unless otherwise indicated. SD: standard deviation; FIM: Functional Independence Measure; NIHSS: National institute of Health Stroke Scale; GCS: Glasgow Comma Scale; CIQ: Community Integration Questionnaire
